# Light-powered phagocytic macrophage microrobot (phagobot): both in vitro and in vivo

**DOI:** 10.1038/s41377-025-01881-3

**Published:** 2025-05-19

**Authors:** Xing Li, Shuhan Zhong, Ting Pan, Jianyun Xiong, Guoshuai Zhu, Yang Shi, Hongbao Xin

**Affiliations:** Guangdong Provincial Key Laboratory of Nanophotonic Manipulation, Institute of Nanophotonics, College of Physics & Optoelectronic Engineering, Jinan University, Guangzhou, 511443 China

**Keywords:** Biophotonics, Optical manipulation and tweezers

## Abstract

Micro/nanorobots based on immune cells show great potential for addressing challenging biological and biomedical conditions. However, their powerful innate immune functions, particularly the phagocytosis capabilities, remain a big challenge to fully leverage with the current designs of immune cell-based microrobots. Herein, we report a light-powered phagocytic macrophage microrobot (phagobot), which is capable of robotic navigation toward specific foreign bio-threats and executing precise phagocytosis of these targeted entities under light control. Without genetic modification or nanoengineering of macrophages, the phagobot’s “wake-up” program is achieved through direct activation of a resting-state macrophage by a tightly focused near-infrared (NIR) light beam. The phagobot exhibits robotic steering and directional navigation controlled by optical manipulation of the extended pseudopodia within the activated macrophage. It can further execute targeted phagocytic clearance tasks via engulfing various foreign bio-threats, including nanoplastics, microbials, and cancer cell debris. Notably, the phagobot can be constructed in a living larval zebrafish through optical activation and manipulation of the endogenous macrophage, which also exhibits controllable navigation and targeted phagocytic capabilities in vivo. With the intrinsic immune functions of macrophages, our light-powered phagobot represents a novel form of intelligent immune cell-based microrobots, holding many new possibilities for precise immune regulation and treatment for immune-related diseases.

## Abstract (web summary)

Light-powered phagocytic macrophage microrobot (phagobot) enables controllable targeted phagocytosis and elimination of biological threats in vitro and in vivo.

## Graphical abstract

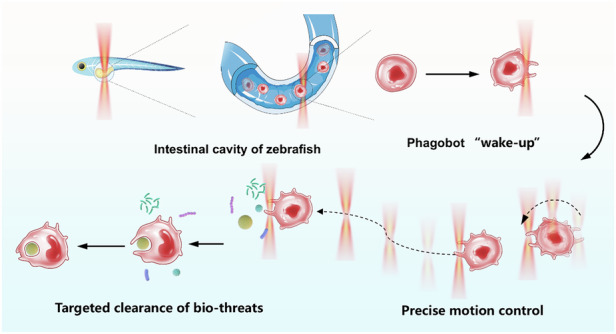

## Introduction

The development of micro/nanorobots has garnered significant attention for accessing complex and deep biological environments to perform various biological tasks due to their small size, precise navigation, and minimal invasiveness^1^. To date, diverse micro/nanorobots have been developed using artificial materials or self-propelled microorganisms like microalgae and bacteria^2–6^. Although these synthetic and biohybrid micro/nanorobots demonstrate excellent mobility control, they are often constructed from artificial materials that are incompatible with physiological environments. Such foreign nature makes these micro/nanorobots to be easily recognized and eliminated by the immune system in the body before they can accomplish their intended biological functions. An innovative solution to overcome this limitation is implementing the innate biological components of the immune system, particularly diverse immune cells, into immune cell-based microrobots (termed as “immunobots”)^7,8^. The development of immunobots facilitates their ability to bypass immune barriers and remain within the body for prolonged durations^9^. Moreover, modulating the immune functions of immune cells in immunotherapy has established a new paradigm for disease treatment, leading to a lot of biomedical breakthroughs^10^. Therefore, it is highly desirable to develop immunobots with robotic control and on-demand immune functionalities for executing biological and biomedical tasks in physiological environments. However, significant challenges remain in finding appropriate actuation methods and real-time regulation of immune cell functions.

Among various immune cells, macrophages play a critical role in the innate immune system. One of the most important features of macrophages is their phagocytosis ability, which enables them to active clearance of foreign pathogens or abnormal cells like cancer cells and apoptotic cells^11^. This capability also allows them to ingest and transport diverse cargos, including large molecules, micro/nanoparticles, and even toxic agents^12^. Based on this unique feature, the current design of macrophage-based microrobots primarily utilizes macrophages as drug carriers with immune-evasion capacity for active and targeted drug delivery to address challenging biological/biomedical conditions, such as cancer treatment and medical imaging^13–16^. The vast majority of these macrophage-based microrobots rely on magnetic actuation by modifying macrophages with magnetic materials due to the advantages of spatiotemporal controllability, fast response, and convenient preparation^7,8,13–15^. However, the interactions between macrophages with materials and drugs may impair the physiological functions of cells, triggering undesired immune responses or toxicity^17^. More importantly, such strategies fail to flexibly control the immune functions of macrophages in vivo. In the current design of immunobots, macrophages can only act as immune escapers or drug carriers, incapable of executing their innate immune functions, such as phagocytosis. Therefore, it is imperative to explore an alternative approach for developing immunobots, in which the immune cells can perform their inherent immune roles.

**Fig. 1:**
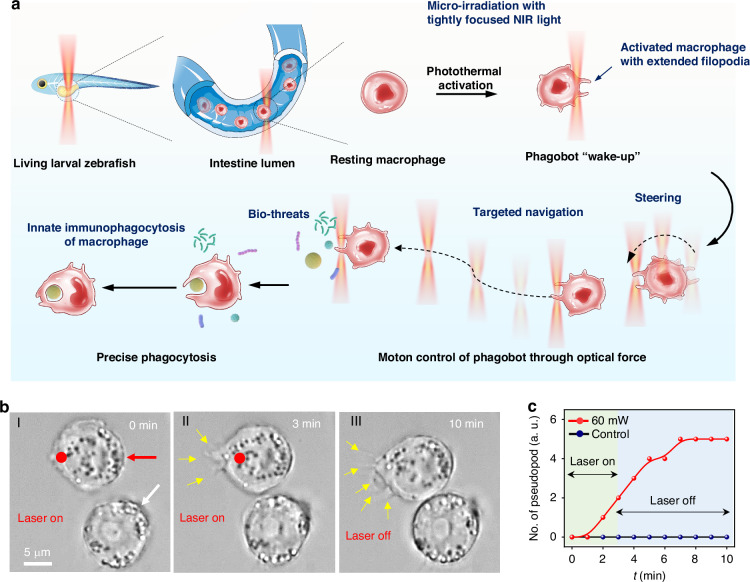
Overview of light-controlled macrophage phagobot. **a** Schematic illustration of light-controlled phagobot for targeted phagocytic clearance of bio-threats. The wake-up program for phagobot is realized by localized optothermal stimulation of a resting macrophage through NIR light micro-irradiation. The steering and navigation of the phagobot can be flexibly controlled via optical manipulation of the extended filopodia in the activated macrophage, and the phagobot can be further controlled to execute immune clearance tasks via phagocytosis of various bio-threats with different sizes. **b** Time-lapse images showing the activation of a resting macrophage through NIR light micro-irradiation. Red dots indicate the position of the NIR light beam, and yellow arrows indicate the extended filopodia in the activated macrophage. **c** Changes in nthe umber of filopodia in (**b**) over time during NIR light micro-irradiation

Thanks to the fantastic multimodal photobiological reactions of light with biological entities, optical techniques have shown great potential for regulating and manipulating living cells in a contactless and noninvasive manner. Optical manipulation based on optical forces offers a flexible approach for direct motion control of living cells with single-cell precision both in vitro and in vivo^18,19^. Moreover, the immune functions of macrophages can be directly regulated by near-infrared (NIR) light^20,21^. Herein, by integrating optical biomodulation and biomanipulation, we report a light-powered phagocytic macrophage microrobot, termed “phagobot,” with controllable phagocytic behavior to effectively target and eliminate specific biological threats both in vitro and in vivo (Fig. 1a). Without cell modification by exogenous artificial materials or gene editing, a resting-state macrophage can be directly activated through precision irradiation with a tightly focused NIR light beam at a wavelength of 1064 nm. This process can be considered as the wake-up program for phagobot. The activated phagobot shows robotic directional navigation by the optical manipulation of the extended pseudopodia within the activated macrophage. The phagobot can then be guided toward targeted positions to perform immune clearance tasks through the phagocytosis of various foreign bio-threats, including nanoplastics, microbials, and cancer cell debris. Notably, the phagobot can be constructed in a living larval zebrafish through direct activation of the endogenous macrophage, which also demonstrates robotic motion control and targeted phagocytic abilities in vivo. This light-powered phagobot serves as a new form of immunobots, with the advantages of both robotic mobility control of micro/nanorobots and immune functions of macrophages.

## Results

### Phagobot activation

The activation and actuation of phagobot were achieved via a focused NIR light system (details see Methods and experimental setup in Fig. S1). A tightly focused laser beam with a wavelength of 1064 nm and a beam diameter of about 2 μm was selected for phagobot activation/actuation due to the weak absorption exhibited by the biological samples at this wavelength. This can reduce the risk of potential photothermal damage induced by light irriadation^22^. The focused laser beam was irradiated on the cell membrane of a target macrophage in vitro or in zebrafish in vivo for multifunctional regulation and manipulation of the macrophage. Resting macrophages typically exhibit a spherical morphology (Fig. 1bI) and are relatively quiescent in function, with no obvious phagocytosis activity^17^. With the micro-irradiation by focused NIR light beam on the cell membrane for 3 min (optical power: 60 mW, pointed by red dot in Fig. 1b and Video S1), pronounced extension of pseudopodia was observed (*t* = 3 min, Fig. 1bII). During this process, the cell morphology gradually changed from a spherical to an extended polygonal shape. After laser turning off, the number of extended pseudopodia continued to increase over the subsequent 7 min (*t* = 10 min, Fig. 1bIII, c). The deformation of macrophages, particularly the extension of pseudopodia, can be considered as an indicator of macrophage activation with enhanced capacity of motility and phagocytosis^23^.

**Fig. 2:**
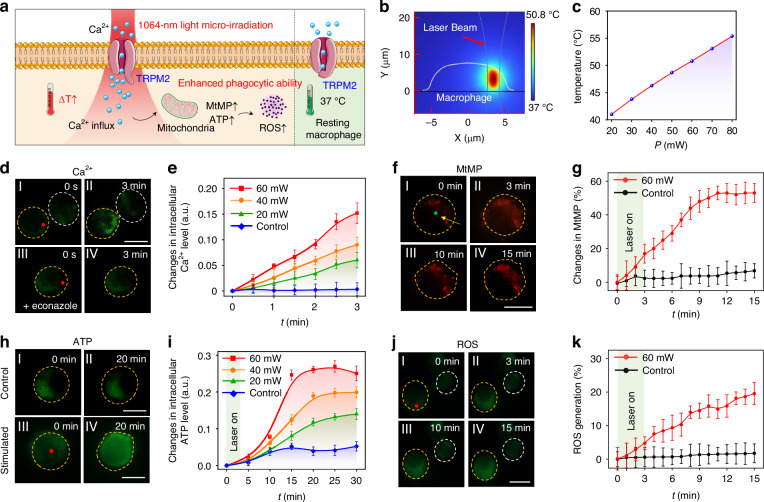
Activation of phagobot through NIR light micro-irradiation. **a** Mechanism of photothermal activation of phagobot by NIR light micro-irradiation. **b** Simulated temperature distribution in a macrophage exposed to NIR light micro-irradiation at an optical power of 60 mW. **c** Calculated temperature of irradiated site in cells at different irradiation power. **d** Intracellular Ca^2+^ levels in macrophages stained with Fluo-4 AM fluorescent probe (I) before and (II) after exposure to NIR light micro-irradiation for 3 min. III and IV, suppression of TRPM2 activation by econazole as negative control. **e** Quantitative statistics of intracellular Ca^2+^ level over time at different irradiation power. **f** Mitochondrial membrane potential (MtMP) analysis of macrophage following NIR light micro-irradiation using fluorescent probe TMRM. The cell was stimulated by micro-irradiation for 3 min, then continuously observed for another 12 min. **g** Quantitative statistics of changes in MtMP over time. **h** Intracellular ATP levels in control and stimulated macrophages with the live-cell ATP dye pCMV-AT1.03. **i** Quantitative statistics of intracellular ATP levels over time during micro-irradiation at different laser power. **j** Intracellular ROS level in a macrophage stained with CM-H_2_DCFDA fluorescent probe. **k** Quantitative statistics of changes in intracellular ROS levels over time at different irradiation power. *n* = 5–6/group. Scale bar for all fluorescent images: 10 μm

After the observation of macrophage morphological change under NIR light micro-irradiation, we further investigated the activation of cellular signaling in macrophages induced by light stimulation. Immune cells, including macrophages, are highly sensitive to alterations in the external environment, particularly to thermal fluctuations^24^. A large number of studies have demonstrated that thermal stimulation can promote macrophage activation and boost their innate immune responses, like phagocytosis and cytokine secretion^25–27^. During the NIR light micro-irradiation, the infrared photon absorption by water and biomolecules can induce photothermal processes^28^, which might facilitate the photothermal activation of the macrophage (Fig. 2a). Thus, we first investigated the photothermal effect of NIR light micro-irradiation on a single macrophage. Figure 2b shows an example of the simulated temperature distribution in a macrophage exposed to the NIR light micro-irradiation with laser power of 60 mW for 3 min (details see Fig. S2). The initial temperature was set at 37 °C, which corresponds to the standard macrophage culture temperature. Upon micro-irradiation for ~20 s, the temperature at the stimulated site is increased rapidly and then reaches a nearly steady state with further irradiation (Fig. S2b). This photothermal-induced temperature increase shows a linear correlation with the applied laser power (Fig. 2c). When the laser power was set to 20 and 60 mW, the stabilized temperature was calculated to be 41.2 and 50.8 °C, respectively. This temperature range is capable to open the thermal-sensitive membrane ion channels in the macrophages^29^. Notably, in the experiments, the macrophages exhibited good viability when exposed to the micro-irradiation at laser power below 60 mW (Fig. S3). This result can be attributed to the fact that the temperature increase was localized around the stimulated site, and the photothermal effect on the entire cell body was negligible. (Fig. 2b). Besides, the increased temperature under these conditions does not cause thermal damage to the cell membrane. Upon increasing the laser power to 80 mW, the calculated temperature was increased to 55.4 °C, a threshold recognized as adequate to cause thermal damage to living cells via disruption of the cell membrane^30,31^. This finding was consistent with our experimental observation of photodamage in macrophages at the same laser power (Fig. S3).

Transient receptor potential melastatin 2 (TRPM2) is a thermosensitive and Ca^2+^-permeable cation membrane channel largely expressed in macrophages (Fig. S4), which facilitates the influx of calcium ions and depolarization of the plasma membrane^29^. It is reported that the activation of TRPM2 is crucial in thermal-regulated phagocytic activity of macrophages^32,33^. We first studied the dynamics of intracellular Ca^2+^ levels in response to NIR light micro-irradiation using Fluo-4 AM, a Ca^2+^ fluorescent probe. As shown in Fig. 2dI, II, the green fluorescence of intracellular Ca^2+^ was rapidly increased in a stimulated macrophage (indicated by yellow circle) after irradiation for 3 min at a laser power of 60 mW as compared to an unstimulated macrophage (indicated by white circle). The intracellular Ca^2+^ level upon light micro-irradiation exhibited a laser-power-dependent increase, with observed increase of 6%, 9%, and 15% at laser powers of 20, 40, and 60 mW, respectively, relative to control cells (Fig. 2e). The intracellular Ca^2+^ fluorescence in macrophage treated with econazole, a TRPM2 inhibitor^34^, remained stable during the light micro-irradiation (Fig. 2dIII, IV), indicating the light micro-irradiation induced Ca^2+^ influx into macrophages through thermal activation of TRPM2 channels.

Reprogramming cellular metabolism plays a crucial role in the activation and functionality of immune cells^35^. Increased cytosolic Ca^2+^ influx in macrophages can trigger a series of cellular responses, especially the changes in mitochondrial activities, which serve as the major ATP supplier and are crucial for regulating macrophage metabolism by supplying the energy required for phagocytosis^36^. We subsequently assessed the mitochondrial activity in macrophages exposed to the optothermal effect induced by light micro-irradiation. The mitochondrial membrane potential (MtMP) serves as a key indicator of mitochondrial functionality, reflecting the processes of reactive oxygen species (ROS) generation and the driving force of ATP production^37^. We first analyzed the MtMP by labeling macrophages with TMRM, a fluorescent probe for MtMP. As indicated in Fig. 2f, the fluorescent signal of MtMP in the stimulated macrophage exhibited a rapid increase during the initial 3 min of micro-irradiation, and continued to rise in a time-dependent manner after laser off. The signal reached a peak value of ~42% relative to unstimulated macrophages at 15 min (Fig. 2g). Since the increased MtMP may contribute to the enhancement of macrophage mitochondrial activity^38^, we further monitored the dynamic changes of ATP concentration in the mitochondria using macrophages transfected with pCMV-Mito-AT1.03 (mito-ATP). As shown in Fig. 2h, compared to the unstimulated macrophage, the macrophage subjected to 3-min NIR light micro-irradiation showed significantly enhanced ATP fluorescent signals both during the stimulation and following the cessation of laser exposure, with continued incubation for an additional 17 min (*t* = 20 min). This observation suggests an increase in mitochondrial activity in macrophages as a result of the micro-irradiation. The up-regulation of ATP generation by micro-irradiation showed a dependency on laser power (Fig. 2i). Specifically, the fluorescence indicating ATP levels exhibited a rapid increase in macrophages subjected to NIR light micro-irradiation for a duration of 3 min. This increase was continued over the subsequent 27 min in the absence of further stimulation, resulting in a final increase of ~14%, 20%, and 25% at laser powers of 20, 40, or 60 mW, respectively, at *t* = 25 min.

Given the critical role of mitochondria in regulating the cellular redox state, the increased MtMP may lead to the rapid production of ROS. This phenomenon is a primary hallmark of the respiratory burst^39^, which is directly linked to the phagocytic capacity of macrophages^40^. Thus, we next investigated the intracellular ROS level in macrophages under NIR light micro-irradiation using ROS-sensitive fluorescent probe CM-H_2_DCFDA. As shown in Fig. 2j, the rapid up-regulation of ROS generation was observed in a macrophage upon a 3-min exposure to micro-irradiation at laser power of 60 mW, with a continued increase after laser turning off for 15 min. In contrast, the time dependence in Fig. 2k shows that the ROS concentration in the unstimulated macrophage persisted at a low level over the entire observation period. Macrophages subjected to micro-irradiation exhibit a steady elevation in ROS production, increased by 17.5% at 15 min as compared to unstimulated macrophages. These results demonstrate the rapid ROS production after NIR light micro-irradiation in macrophages, which is fundamental for macrophages to eradicate foreign threats through the respiratory burst. Together, the micro-irradiation with NIR light triggers macrophage activation through mechanisms involving optothermal activation of thermosensitive membrane ion channels, leading to Ca^2+^ influx, which boosts mitochondrial activity and ROS production (Fig. 2a). Additionally, the extension of pseudopodia (Fig. 1b) temporally correlates with the observed ROS burst and enhanced mitochondrial activity, establishing a direct causal link between the optothermal-triggered signaling cascade and the resulting morphological activation. This process can be considered as the wake-up program of the phagobot from resting state to activated state to respond to foreign bio-threats through increased phagocytosis.

It is noteworthy that although an increase in laser power from 20 to 60 mW resulted in enhanced activation of macrophages, further increasing the laser power to 80 mW can induce significant photodamage to the cells (Fig. S3). The cell viability assay demonstrated that when the laser power was maintained below 60 mW, there was no significant impact on macrophage viability even with prolonged light exposure (Fig. S4). However, when the laser power exceeded 80 mW, macrophages exhibited pronounced cell shrinkage and membrane blebbing (*t* = 30 min). Therefore, after a thorough evaluation of activation efficiency and cell viability, a laser power of 60 mW demonstrates the optimal activation efficiency for the phagobot.

### Motion control of phagobot

**Fig. 3:**
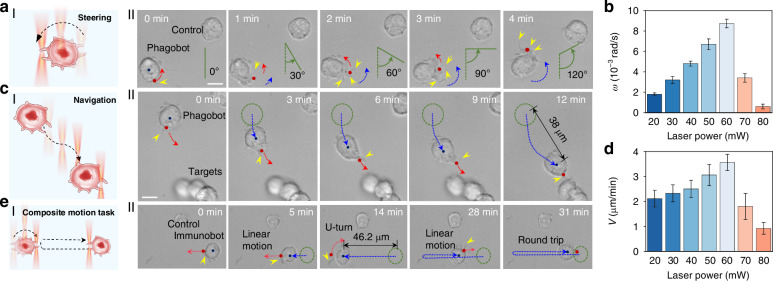
In vitro motion control of phagobot. **a** Phagobot steering. I: schematic illustration of controllable rotation of phagobot steered by programmed annular scanning focused light beam. II: time-lapse image sequences of the steering/rotation of the phagobot. Red dots indicate the position of light beam, red dashed curves indicate the predesigned light beam trajectory, blue lines indicate the steering angle, and yellow dots point to the extended filopodia. **b** Angular velocity (*ω*) of phagobot steering/rotation regulated by varying optical power. *n* = 5–6/group. **c** Phagobot navigation. I: schematic illustration of directed navigation of phagobot controlled by programmable optical force. II: time-lapse image sequences of directed navigation of the phagobot toward a targeted cell. Green dashed circles and blue dashed lines indicate the initial position and the moving trajectory of the phagobot, respectively. **d** Quantitative statistics of linear motion velocity of phagobot as a function of laser power. *n* = 5–6/group. **e** Composite motion task of directional steering and navigation. I: schematic illustration of directional steering and navigation of phagobot controlled by programmed composite light beam. II: time-lapse image sequences of the round-trip movement with U-turn action of the phagobot. Scale bar: 10 μm

Controllable actuation and navigation of the phagobot are crucial for executing different on-demand immune tasks, such as targeted phagocytosis of foreign bio-threats and killing of cancer cells. Macrophages are known to migrate toward mechanical stimuli, wherein filopodia play a crucial role in sensing mechanical forces and driving cellular migration along the orientation of the guiding stimulus^41,42^. On this basis, the steering and navigation of the phagobot were optically controlled through manipulating the extended filopodia of the activated macrophage via optical force along a predetermined route in a contactless and noninvasive manner. Firstly, the steering of the phagobot was realized by manipulation of the filopodia via moving the light beam in a designed annular trajectory centered on the cell (Fig. 3aI and Video S2). As indicated in Fig. 3aII, under NIR light micro-irradiation at the cell boundary (laser power of 60 mW), the phagobot was first activated to stretch out filopodia (*t* = 0 min). A laser beam was then applied to the filopodia to exert optical force on them (*t* = 2–8 min). These filopodia were found to act as navigational guides for the phagobot, which can sense and move toward the optical force stimuli, thus driving the phagobot to undergo a rotational motion. The rotation angle of the phagobot can be precisely controlled by the scanning trajectory of laser beam, achieving a rotation angle of ~120° at *t* = 4 min. The rotation speed of the phagobot was mainly determined by the laser power (Fig. 3b). When the laser power was below 60 mW, the rotation speed increased with the increase of laser power, with a maximum value of about 8.7 × 10^−3^ rad s^−1^ at a laser power of 60 mW. However, beyond this threshold, a significant decline in rotation speed was observed with further increases in laser power, attributed to the decreased cell activity resulting from photodamage exerted on macrophages by the relatively high laser intensity. Therefore, by utilizing the wireless and adaptable manipulation of optical force, the steering of the phagobot could be precisely achieved with controllable rotation angle and speed.

In addition to the controllable steering capability, the orientational navigation of the phagobot can also be precisely controlled. To achieve this, the filopodia of the activated macrophage were manipulated through optical force generated by the tightly focused NIR laser light to drive the phagobot migrating along a predefined linear trajectory (Fig. 3cI and Video S3). Figure 3cII illustrates the directed navigation of the phagobot toward a targeted location guided by optical force. Initially, the macrophage was activated by light micro-irradiation (indicated by the red dot) at the edge of the target cell, resulting in the formation of filopodia (yellow arrow) at *t* = 0 min. Subsequently, by optically manipulating the extended filopodia with a laser beam, the navigation of the phagobot was precisely controlled (moving trajectory indicated with blue curve in Fig. 3cII). Upon the stimulation by the optical force, the extended filopodia exhibited a tendency to move toward the laser beam, thereby driving the phagobot to migrate along the moving path of the laser beam. This process enabled the phagobot to reach the targeted location at *t* = 12 min, achieving a total displacement of about 38 μm. The migration velocity (*V*) of the phagobot was modulated by varying the laser power (Fig. 3d). An increase in laser power resulted in a gradual increase in migration velocity, reaching a maximum value of 3.4 μm min^−1^ when the laser power was 60 mW. However, a further increase in laser power led to a significant decline in velocity due to the photodamage to cells. The optical force exerted on the pseudopodia of the phagobot was also roughly estimated by an optically trapped bead (details see Section 1.5 and Fig. S6a in Supplementary Information). The estimated optical force exhibits an upward trend with the increase in laser power (Fig. S6b). Specifically, as the laser power is increased from 20 to 80 mW, the corresponding optical force is increased from 25.7 to 38.6 pN. This value is consistent with findings from other studies investigating the optical force applied to pseudopodia^43,44^.

To demonstrate the navigation flexibility of the phagobot, the phagobot was controlled to execute composite motion tasks by integrating the two motion modes of steering and navigation (Fig. 3eI). As illustrated in Fig. 3eII, upon activation, the phagobot underwent a linear motion guided by a linearly manipulated light beam, with a navigation distance of 46.2 μm at *t* = 14 min. Subsequently, the movement mode of the light beam was altered to follow a designed annular trajectory centered on the cell, guiding the phagobot to do a U-turn motion toward its original direction. Thereafter, the movement mode of the light beam was reverted to a linear motion, enabling the phagobot to return to its initial position (*t* = 31 min). These results demonstrate that the light-powered phagobot successfully executed complex trajectory movements with flexibility.

### Targeted clearance of bio-threats by phagobot in vitro

**Fig. 4:**
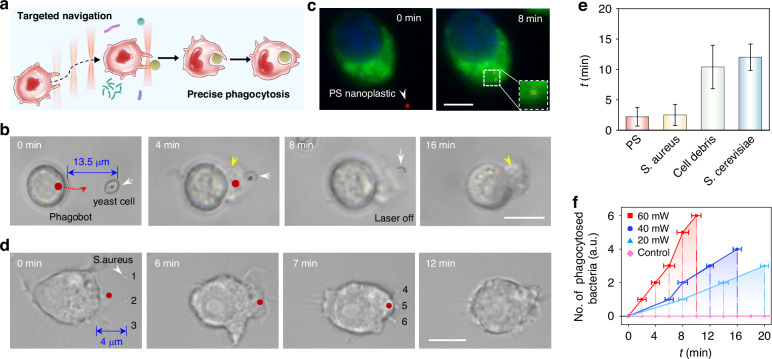
Execution of targeted phagocytic tasks. **a** Schematic showing targeted phagocytosis of foreign bio-threats by light-controlled phagobot. **b** Time-lapse image sequences showing the phagocytosis process of a yeast cell by phagobot. Red dots indicate the position of light beam, red dashed curves indicate the predesigned light beam trajectory, white and yellow arrows indicate the targeted yeast cell and extended filopodia in the phagobot, respectively. Scale bar: 10 μm. **c** Fluorescent images showing the phagocytosis of a 500-nm polystyrene (PS) nanoparticle by phagobot. Red: PS nanoparticle, blue: cell nucleus of macrophage, green: actin cytoskeleton. Scale bar: 5 μm. **d** Sequential phagocytosis of multiple *S. aureus* bacteria by phagobot in a programmable manner. **e** Average rate of engulfment (min ± SD) of a single bio-threat of different sizes by the phagobot. *n* = 5–6/group. Scale bar: 10 μm. **f** Number of phagocytosed bacteria by phagotbot over time by varying laser powers. *n* = 5–6/group

Precisely regulating macrophages to execute their immune functions, such as the elimination of foreign biological threats through phagocytosis, is of significant importance; however, it also presents considerable challenges to precisely regulate the immune state of macrophages. The controllable activation and navigation capabilities make our light-powered phagobot a promising candidate for targeted phagocytic clearance of bio-threats (Fig. 4a). Microorganisms are the most typical foreign bio-threats to human bodies, causing various illnesses, such as pathogens. We first investigated the potential of the phagobot for targeted phagocytosis of microorganisms. We used yeast cell *S. cerevisiae* as an example, which possesses an average diameter of about 3–5 μm (Fig. 4b). To achieve targeted clearance of a yeast cell located 13.5 μm away from the macrophage, the macrophage was firstly activated by light micro-irradiation (laser power: 60 mW) to transform into a controllable phagobot (*t* = 0 min). Subsequently, the phagobot was navigated toward the targeted yeast cell (*t* = 4 min). Upon arrival at the targeted yeast cell, the light was turned off, and the phagobot maintained strong phagocytic capability, which phagocytosed the yeast cell through its extended filopodia within an additional 12 min (*t* = 16 min). These data indicate that the phagobot successfully navigated to the designated location and effectively phagocytosed targeted microorganisms.

Micro/nanoplastics have become another non-negligible bio-threat due to the widespread usage and improper management of plastics. The targeted phagocytic capability of phagobot was also demonstrated on polystyrene (PS) nano-sized plastic particles with a diameter of 500 nm (Fig. S7a and Video S4). To better observe the phagocytosis of phagobot, macrophages were labeled with fluorescent probes for live-cell imaging of the cytoskeleton and cell nuclei (Fig. 4c). The activated phagobot was navigated by optical force to phagocyte a targeted 500-nm PS nanoparticle labeled with red fluorescence. And the macrophage engulfment of PS nanoparticle was demonstrated by the colocalization of the cytoskeleton and nanoparticle, which was depicted in yellow (overlap of green and red fluorescence). Furthermore, the phagobot can be directed to sequentially engulf multiple bio-threats in a programmable manner. As indicated in Fig. 4d, three *S. aureus* bacterial cells, each with a diameter of about 0.8 μm, were aligned parallel to the phagobot at an interval of 4 μm. Upon light activation and optical manipulation, the phagobot migrated toward the bacterial cells and successfully engulfed them within 6 min (*t* = 6 min). Subsequently, another three *S. aureus* bacterial cells were placed beside the phagobot (*t* = 7 min), and it proceeded to engulf these bio-threats within an additional 3 min (*t* = 10 min). In addition to microorganisms and plastic nanoparticles, the macrophage immunobot was also able to engulf micrometer-scale-sized cancer cell debris of HeLa cells with irregular shapes (Fig. S7b). Additionally, the phagobot can also simultaneously phagocytose multiple bio-threats, thereby demonstrating its high precision and efficiency in phagocytic capabilities (details see Section 1.7 and Fig. S8 in Supplementary Information).

The phagocytic rate of phagobot was significantly correlated with the size of the targeted bio-threats (Fig. 4e). Specifically, the phagocytosis of smaller particles, with a diameter less than 1 μm, such as PS nanoparticles and *S. aureus* bacterial cells, took about 2 min. In contrast, the phagocytosis of larger particles, including *S. cerevisiae* yeast cells and cancer cell debris, required about 10 min, due to the fact that more energy is required for phagocytosis of larger particles. These results demonstrated that the light-powered phagobot was capable of migrating to designated locations to phagocytose different bio-threats of diverse sizes. Notably, the phagocytic ability of the phagobot can be modulated by altering the laser power (Fig. 4f). At a laser power of 60 mW, the phagobot consecutively engulfed six *S. aureus* bacterial cells within about 10 min. A reduction in laser power resulted in a prolonged duration for the phagobot to engulf fewer bacterial cells. Specifically, the phagobot took about 16 min to engulf four bacterial cells at a laser power of 40 mW, and about 20 min to engulf three cells at 20 mW. These findings suggested that higher laser power can enhance the phagocytic activity of the phagobot, aligning with the results observed in the activation studies of the phagobot.

### Construction of phagobot in vivo and targeted elimination of cell debris

**Fig. 5:**
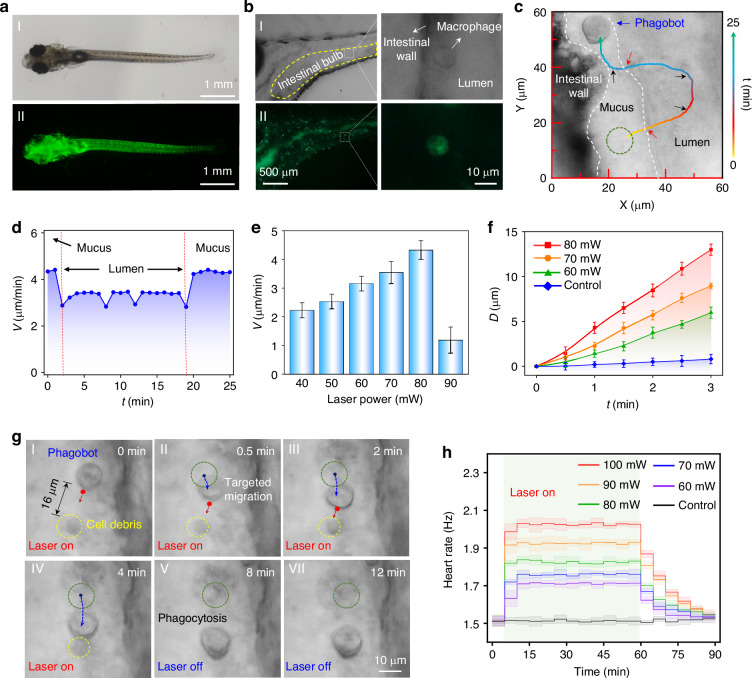
Construction and navigation control of phagobot for targeted phagocytosis in a living larval zebrafish. **a** Optical microscopy (I) and fluorescence images (II) of larval zebrafish with fluorescence-labeled macrophages. Scale bar: 500 μm. **b** Optical microscopic images for observing single fluorescence-labeled macrophages in the intestinal cavity tissues of larval zebrafish. Scale bar: 10 μm. **c** Navigation of a phagobot along a complex trajectory in the intestinal cavity tissue. Red dots and red arrows indicate the position and moving direction of light beam, respectively. Green dashed circles and blue dashed lines indicate the initial position and the moving trajectory of the phagobot, respectively. **d** Migration velocity of phagobot over time in (**c**). In vivo **e** navigation velocity and **f** locomotion distance of the phagobot as a function of laser power. *n* = 5–6/group. **g** Optical microscopic images for navigating the phagobot to eliminate targeted cell debris. Yellow dashed circles indicate the targeted cell debris. **h** Measured heart rates of zebrafish stimulated by various laser powers. *n* = 5 hearts

Encouraged by the in vitro results, we further elucidated the on-demand, precise activation and actuation of the phagobot and its potential phagocytic clearance of bio-threats in vivo. Larval zebrafish were selected as the in vivo animal model due to their transparency and high genetic resemblance to humans^45^, which allows for light-based manipulation (Fig. 5aI). This transparency also allowed for the clear identification of macrophages through fluorescence labeling (Fig. 5aII). To turn an endogenous macrophage into a phagobot in living larval zebrafish, the NIR light beam was precisely focused on the periphery of a macrophage located in the intestinal bulb (Fig. 5b), an area abundant with macrophages during the developmental stages of larval zebrafish. The morphological changes observed in macrophages following NIR light micro-irradiation suggest the activation of phagobots in vivo (Fig. S9). Subsequent to this activation, the intelligent behavior of the phagobot was conducted through optical force in a programmable manner. Similar to in vitro motion control, the phagobot successfully executed a composite motion task involving directional steering and navigation in vivo with a complex moving trajectory (Fig. 5c and Video S5). During the experiment, different tissue regions in the larval zebrafish can be roughly distinguished according to different brightfield imaging contrast under the same visual field (distinguished by white dashed lines as intestinal wall, mucus, and lumen). The orientation of filopodia in the activated macrophage was manipulated to guide the phagobot along a predetermined trajectory, allowing for controlled turning at designated locations (specifically, *t* = 8, 12 and 21 min, as indicated by black arrows in Fig. 5c). The phagobot navigated from the mucus to the lumen, and subsequently returned to the mucus (indicated by red arrows in Fig. 5c), indicating the phagobot can navigate through different tissue regions under light control. The migration velocity exhibited slight variations across different tissue regions due to the varying tissue densities (Fig. 5d). Furthermore, as laser power increased, the velocity was also increased, driven by the enhanced optical force (Fig. 5e). When the laser power reached 80 mW, the velocity reached a maximum value of 4.3 μm min^−1^, which was 3.6 times over the spontaneous migration speed of macrophages in vivo (~1.2 μm min^−1^)^46^. However, as the power further increased to 90 mW, due to the photothermal damage (Fig. S11), the velocity was decreased. For an equivalent time, the navigation distance of phagobot controlled at 80 mW was 1.3 times larger than that at 60 mW (Fig. 5f). It should be noted that the laser power required for in vivo light actuation of the phagobot is ~1.3 times higher than that employed in vitro. This increase is likely attributable to the unavoidable tissue scattering, necessitating higher laser power to achieve effective activation and manipulation of the phagobot in vivo.

After the in situ activation and flexible navigation of the phagobot in vivo, its targeted phagocytic ability was further studied. As indicated in Fig. 5g, an activated phagobot was guided to navigate toward a cell debris (as indicated by the yellow circle) with a distance of 16 μm at *t* = 0 min. With the assistance of optical navigation, the phagobot was driven to navigate toward the target (*t* = 0.5 min and 2 min). At *t* = 4 min, upon reaching the cell debris, the phagobot began to execute its phagocytic task. After an additional 8 min (*t* = 12 min), the cell debris was completely engulfed by the phagobot. Consequently, through the active and precise regulation of the phagobot in vivo, it can execute the targeted phagocytic tasks at the predesigned time and location. The above results indicate that the phagobot is capable of navigating and executing complex tasks within the intestinal cavity, an environment characterized by its complex and highly variable morphology, including luminal folds, heterogeneous mucus distribution, and continuous peristaltic movements. These results indicate the in vivo applicability of the phagobot. Additionally, the phagobot exhibits better performance for the situation of in vivo than in vitro, with improved navigation speed and phagocytic ability. This enhanced in vivo performance might arise from the differences in macrophage behavior between in vitro 2D culture conditions and in vivo 3D physiological environments^47^. Macrophages in in vitro 2D culture conditions adhere to rigid planar substrates and are immersed in artificial culture medium. In contrast, in vivo macrophages interact with various cell types, extracellular components, and soluble factors that collectively regulate their behavior. These factors may promote a more natural, primed state of macrophages, allowing for enhanced sensitivity to external stimuli and better motility^48^. These intrinsic advantages of the in vivo 3D milieu collectively enable more efficient optothermal actuation and manipulation of phagobot within living organisms.

It should be noted that the biological safety for in vivo applications is very important. In this study, phagobot activation and navigation were performed using a wavelength of 1064 nm, which is characterized by low absorption in tissues. We have systematically analyzed the in vivo biological safety of the phagobot system at cellular and organism levels (Figs. 5h, S10 and S11). During the experiments, the heart rates of larval zebrafish were observed to slightly increase upon light stimulation and then gradually return to their normal state after light was turned off (Figs. 5h and S10). The observation of the larval zebrafish’s heart rate confirms the biological safety of the phagobot system at the organism level. Although the zebrafish remained alive at each laser power, higher laser intensities (above 90 mW) can cause significant photodamage to the irradiated cells (Fig. S11). This observation is consistent with the in vivo performance of phagobot at different laser powers (Fig. 5e). Therefore, at laser power below 80 mW, our light-powered phagobot showed good biocompatibility and minimal invasiveness in vivo.

## Discussion

In summary, a light-powered phagocytic macrophage microrobot (phagobot) was developed both in vitro and in vivo. This phagobot demonstrates robotic motion control and phagocytic capabilities, which can be activated on demand to precisely target and eliminate specific biological threats. With a localized optothermal stimulation via NIR light micro-irradiation, a resting macrophage was remotely activated to turn into a phagobot with increased Ca^2+^ influx, enhanced mitochondrial activity, and ROS production. The activated phagobot exhibits the capability to execute various motion tasks, such as steering and directional navigation, which can be flexibly navigated along arbitrary complex path with controllable velocity. Combining the high-precision spatiotemporal control by light and the robust phagocytic capabilities of macrophages, the phagobot demonstrates targeted elimination of various foreign bio-threats of different sizes both in vitro and in vivo in a living zebrafish. Furthermore, the construction and operation of phagobot in living larval zebrafish highlight its biocompatibility, minimal invasiveness, and potential for precise immune modulation in complex biological environments.

Precise regulation of immune cell function and behavior is crucial for enhancing effective immune responses and advancing immunotherapy. While genetically engineered macrophages demonstrate enhanced tumor-targeting and tumor-killing capabilities compared to their native counterparts^49,50^, their applications face challenges including time-consuming preparation and off-target toxicity risks^51^. Magnetically propelled immunobots offer improved controllability with relatively high velocity (25–80 μm s^−1^)^7,13,15^, yet concerns exist regarding mechanical stress-induced functional alterations in macrophages under strong force fields. Alternative functionalized nanoparticle-based microrobots can also be applied for immune cell regulation^52^. But their intrinsic foreign nature presents unavoidable biocompatibility challenges^53^. Current optically driven bio-microrobots predominantly utilize flagellated microorganisms, such as microalgae and bacteria^4,5,54,55^. By leveraging their intrinsic motility, the navigation velocity of these bio-microrobots can be relatively high (2–60 μm s^−1^)^4,5,54,55^. Instead of microorganisms, our phagobot was constructed based on mammalian cells, which widely exist in our human body. In contrast to conventional bio-microrobots, which rely on external forces for the mechanical propulsion of the entire cell, our approach employs optical force to guide pseudopodia-directed movement. Although slower (*V*_*max*_ = 4.3 μm min^−1^ in vivo), this approach provides a direct way to actuate living cells, which significantly preserves the macrophage’s inherent motility and immune functionality. Without genetic modification or synthetic components, the phagobot achieves single-cell targeting precision, which can hardly be achieved by the current microrobots with bulk manipulation. Furthermore, it eliminates biocompatibility risks and mechanical disruption associated with magnetically actuated immunobots. To further enhance the navigation velocity of the phagobot, we propose a multimodal actuation strategy by combining optical actuation with magnetic/ultrasound-driven navigation for rapid long-distance navigation. However, future studies must be carried out to rigorously assess the potential nanoparticle-induced immunophenotypic alterations and mechanical stress effects on macrophage functionality during magnetic/ultrasound field actuation.

We have successfully demonstrated the actuation and manipulation of the phagobot using NIR light in vivo within living zebrafish. However, the inherent limited penetration of light in deep tissues, primarily due to scattering and absorption, impedes its further applications in the deep tissue of larger mammals. The integration of advanced optical techniques specifically addressing this challenge, such as flexible optical fibers^56^, two-photon excitation^57^, and the use of optical clearing agents^58^, may provide viable solutions for the deep-tissue operation of the phagobot. Although optical force has been extensively utilized in cell manipulation and the development of light-driven cellular robots, its capacity to regulate cellular functions remains rarely explored. This is because the mechanical stimuli generated by optical force are typically too weak (generally piconewton scale) to induce cellular signal transduction. For instance, mechanosensitive membrane ion channels require mechanical forces on the nanonewton scale for activation^59,60^. Our integrated strategy of optical biomodulation and manipulation offers a promising approach and novel concept for the development of light-powered living cell robots, facilitating more profound regulation of cellular functions. Furthermore, other phagocytic innate immune cells, such as neutrophils and dendritic cells, exhibit analogous functional characteristics and sensitivity to external stimuli as macrophages^24^. This suggests that the cell types employed in the development of phagobot could be expanded beyond macrophages to these immune cells. Additionally, exploring the modulation of more immune functions beyond phagocytosis, such as cytokine secretion and antigen presentation, may broaden the biomedical applicability of the light-powered phagobot. Its potential applications range from managing inflammatory diseases to treating infections and cancers, laying the foundation for future advances in precision medicine and immune engineering.

## Materials and methods

### Cell culture

RAW264.7 macrophages were procured from the China Type Culture Collection. Unless otherwise stated, cell culture reagents were purchased from Gibco (ThermoFisher Scientific, USA) and Hyclone (ThermoFisher Scientific, USA). Macrophages were cultured in high glucose DMEM containing GlutaMAX and sodium pyruvate, supplemented with 10% fetal bovine serum, 100 U/mL penicillin, and 100 μg/mL streptomycin. Cells were incubated at 37 °C in a humidified atmosphere consisting of 5% CO_2_. Before experiments, macrophages were seeded onto 35-mm glass-bottom Petri dishes, and then the experiments were conducted in the living cell workstation (37 °C, 5% CO_2_).

### Experiment setup

The experiment setup was constructed around a focused NIR light system (Tweez250si, Aresis Co., Ltd., Slovenia) for a flexible micro-irradiation and optical manipulation (see Fig. S1 for an overview). The system consisted of an inverted fluorescence microscope (Nikon Eclipse Ti-U), a continuous-wave solid-state laser operating at 1064 nm, an acousto-optic deflector (maximum switching rate: 100 kHz) as a spatial light modulator to control the laser beam, and a CMOS camera for real-time imaging. The laser was focused to a Gaussian shape through a 60× water-immersion inverted objective (DIC, NA = 2.0, Nikon) before interacting with the cell samples. The operating laser power ranged from 0 to 250 mW. Illumination light from a halogen source (D-LH/LC:12 V, 100 W) was focused through a condenser onto the sample for brightfield imaging. The experiment was monitored and captured in real-time using a computer-interfaced CCD camera (maximum frame rate: 100 Hz) to perform visual control and image acquisition.

### Phagobot activation and navigation

Macrophages were plated on 35-mm glass-bottom Petri dishes in complete medium at 37 °C with 5% CO_2_. Optothermal activation of phagobot employed a static focused laser beam applied to the cell membrane for 3 min to ensure localized temperature elevation reached the threshold for macrophage activation. The changes in cell morphology and pseudopodia elongation were observed through the optical microscope. After phagobot activation, the laser beam was moved to the extended pseudopodia for optical force-based motion control. The beam position was adjusted dynamically to achieve precise optical trapping and manipulation of macrophage pseudopodia for real-time control over the movement of the phagobot.

### Immunofluorescent staining of TRPM2

Cells were fixed with 4% paraformaldehyde in 0.1 M PBS (pH 7.4) for 30 min at room temperature. Then, the cells were incubated at 4 °C overnight with a primary antibody specific to TRPM2 (Abcam, ab11167; 1:200). After washing and blocking, the cells were incubated with an Alexa Fluor 647-conjugated secondary antibody (Abcam, ab150079; 1:200) for 1 h at room temperature. The samples were observed under a Nikon Ti2-A inverted fluorescence microscope.

### Intracellular Ca^2+^, ROS level, and mitochondrial membrane potential analysis

Before experiments, macrophages were transferred into 35 mm glass-bottom dishes and cultured for 24 h. Then, the growth medium was removed and the macrophages were incubated at 37 °C for different time intervals in DMEM medium containing one of the following fluorescence probes: Fluo-4 AM (Invitrogen™, USA) for monitoring Ca^2+^ cellular level (green fluorescent, 494/506 nm, 5 µM), CM-H_2_DCFDA (Invitrogen™, USA) for examing the intracellular ROS level (green fluorescent, 504/525 nm, 2 µg mL^−1^), Image-iT^TM^ TMRM reagent (Invitrogen^TM^, USA) as indicator of MtMP (red fluorescent, 548/574 nm, 1 µg mL^−1^), according to manufactor’s instructions and previous reports. After incubation with each probe, the stained macrophages were thoroughly washed with colorless DMEM. The colorless DMEM was also used in the optical activation and manipulation experiments. Changes in the probe fluorescence signal (caused by Ca^2+^ influx, ROS level, or change in MtMP) were detected and imaged using the inverted fluorescence microscope. The fluorescence intensity was calculated from digitized images with ImageJ software. Econazole, a TRPM2 inhibitor, was dissolved in dimethyl sulfoxide and subsequently added to macrophage cultures at a final concentration of 30 µM before the application of fluorescent labeling of intracellular Ca^2+^.

### Mitochondria ATP analysis

The plasmid pCMV-Mito-AT1.03 (Beyotime, Shanghai, China) was applied to monitor the mitochondrial ATP levels in living macrophages. By using a CMV promoter, this construct directs the expression of the AT1.03 protein tagged with a mitochondrial targeting sequence, enabling dynamic observation of ATP concentration changes in mitochondria. Following the manufacturer’s guidelines, macrophages were cultured in 35-mm glass-bottom Petri dishes and subjected to transfection with pCMV-Mito-AT1.03 using the Lipofectamine 3000 reagent (ThermoFisher, USA) over 48 h. Then, the cells were manipulated and observed with the focused NIR light system equipped with an inverted fluorescence microscope.

### Live-cell imaging of the cytoskeleton and cell nuclei

Living macrophages were transfected with Actin-GFP using DNA plasmids pCMV-LifeAct-TagGFP2 (Miaoling Biology, China) and LipofectamineTM LTX (ThermoFisher Scientific, USA) according to the manufacturer’s instructions and previous reports. After 24–48 h, the transfected cells were seeded onto substrates for the experiments. Nuclei were visualized with Hochest 33258 staining (ThermoFisher Scientific, USA).

### Animal experiments

All in vivo experiments were performed in compliance with the Laboratory Animal Ethics Committee of Jinan University. The larval zebrafish, aged 6 days post-fertilization (dpf), were procured from the Nanjing Eze-Rinka Biotechnology Co., Ltd. (Nanjing, China). Following established protocols, the larval zebrafish were housed in a clean tank and maintained under a 14-h light/10-h dark cycle at a temperature of 28.5 °C. The transgenic zebrafish line utilized was Mpeg1: GFP, which facilitates the in vivo visualization of macrophages through fluorescence labeling. Before experiments, the larval zebrafish were anaesthetized by incubation with MS-222 at a concentration of 0.08 mg mL^−1^ for 5 min and subsequently immobilized on agarose slices. Subsequently, the sample was placed on a motorized translation stage to ensure accurate positioning within the *x*–*y* plane, facilitating both observation and optical manipulation. All experiments were conducted in compliance with the ethical standards set forth by the Laboratory Animal Ethics Committee of Jinan University.

### Data analysis

Data were collected from a minimum of three independent experiments and presented as mean ± standard deviations (SD). Statistical analyses were conducted using Origin software, and the images were processed with ImageJ software.

## Supplementary information

Supplementary Information

Video S1. Activation of a resting macrophage through NIR light micro-irradiation

Video S2. Controllable steering of phagobot in vitro

Video S3. Navigation of phagobot in vitro

Video S4. Targeted phagocytosis by phagobot

Video S5. Navigation of a phagobot in the intestinal lumen in vivo

## Data Availability

The data that support the findings of this study are available from the corresponding author upon reasonable request.
