# Supplementary material for: Light-powered phagocytic macrophage microrobot (phagobot): both in vitro and in vivo
Source: Light Sci Appl. 2025 May 19;14:202. doi: 10.1038/s41377-025-01881-3 (PMC12086205; doi:10.1038/s41377-025-01881-3)
Supplement: Supplementary file 1 — Supplementary Information [file 41377_2025_1881_MOESM1_ESM.pdf]

Supplementary Information for

Light-powered phagocytic macrophage microrobot (phagobot): both in vitro and in vivo

Xing Li, Shuhan Zhong, Ting Pan, Jianyun Xiong, Guoshuai Zhu, Yang Shi, and Hongbao Xin**

Guangdong Provincial Key Laboratory of Nanophotonic Manipulation, Institute of Nanophotonics, College of Physics & Optoelectronic Engineering, Jinan University, Guangzhou 511443, China

*To whom correspondence may be addressed:

E-Mail: tingpan@jnu.edu.cn; hongbaoxin@jnu.edu.cn

This word file includes:

1. Supplementary Figures

Fig. S1 to S11

2. Description of Supplementary videos

Video S1 to S5

3. References

1. Supplementary Figures

1.1 Experimental setup of the focused near infrared (NIR) light system

As shown in Fig. S1, the focused NIR light system consists of an inverted optical microscope (Nikon Eclipse Ti-U), a continuous-wave solid-state laser operating at an optical wavelength of 1064 nm, a personal computer (PC) interfaced acousto-optic deflector (AOD) system used as spatial light modulator (SLM) to control the laser beam, and a PC interfaced CMOS camera for real-time capture of the image and videos. A 60× water-immersion inverted objective is used for laser beam focusing.

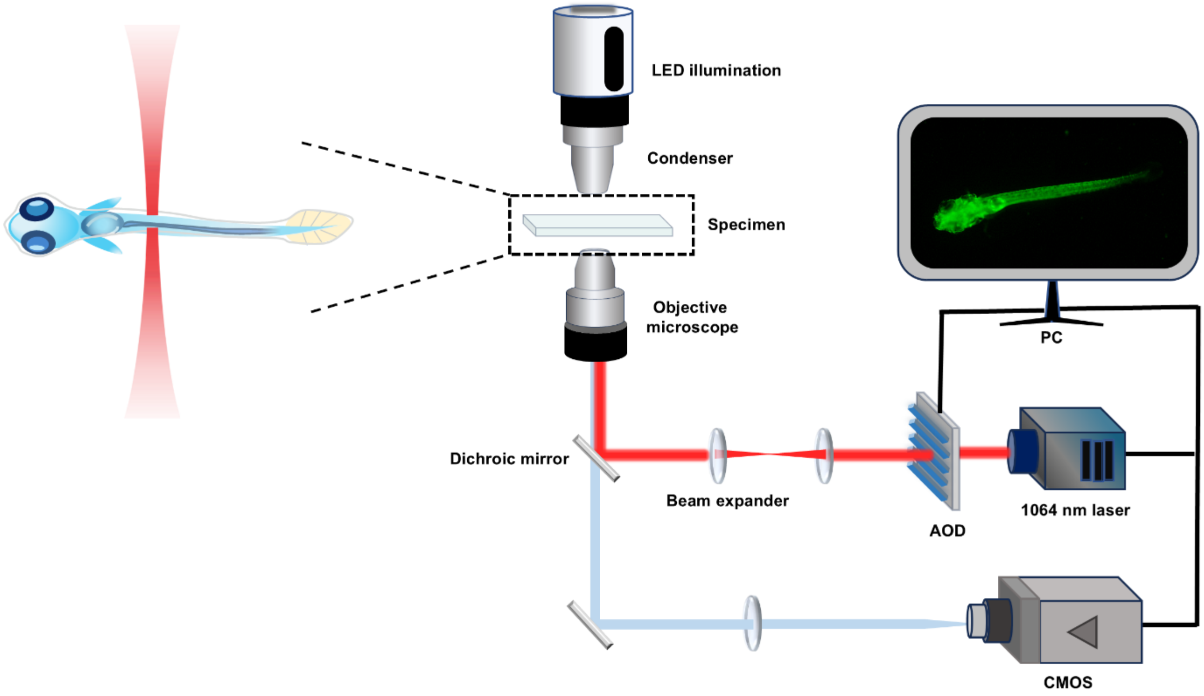

Figure S1. Schematic diagram of the experimental setup of the focused near infrared (NIR) light system.

1.2 Simulation of photothermal effects of NIR light micro-irradiation on a single macrophage

In the simulation, COMSOL Multiphysics, a finite element analysis software, was employed for computational modeling. The Heat Transfer Module was used to model the temperature field distribution in the macrophage exposed to NIR light micro-irradiation. The geometry of the macrophage was approximated as a flattened spheroid with an adhesive surface to substrate, mimicking the typical shape of a macrophage in standard 2D adherent culture. The diameter of the macrophage and laser beam was set to 12.5 μm and 2.5 μm, respectively. Corresponding to standard macrophage culture conditions, the boundary temperature, and the initial temperature for both the macrophage and the surrounding medium was set to 37 °C. The simulation was performed for a time range of 0 to 180 s, as the temperature increase at the stimulated site stabilized within this time. A "Regular" mesh resolution was used to balance computational accuracy and efficiency.

The thermal properties of the macrophage and the surrounding medium were approximated to those of biomass and water, respectively.[1] Specifically, the density (ρ), specific heat capacity (C_p) and thermal conductivity (k) of macrophages were set as 1100 kg/m^3, 3600 J kg^{-1} K^{-1} and 0.55 W m^{-1} K^{-1}, respectively. And the ρ, C_p and k of surrounding medium were set as 998 kg m^{-3}, 4186 J kg^{-1} K^{-1} and 0.6 W m^{-1} K^{-1}, respectively. The model assumed that the culture medium was stationary, as the influence of fluid flow on heat transfer was considered negligible in this study. Heat Transfer in solids and fluids was selected to account for heat exchange between the macrophage (solid) and the surrounding culture medium (fluid), but the Non-Isothermal Flow setting was not enabled due to the stationary nature of the medium.

The heat source (Q) was defined using a Gaussian distribution to model the laser irradiation:

$$Q = \frac{\eta P}{\pi w_0^2} \exp\left(-\frac{r^2}{w_0^2}\right)$$

where P is the laser power, w_0 is the radius of the laser beam, r is the radial distance from the center of the laser beam, $\eta = 1$ (assuming full absorption of the laser energy by the cell). To describe the temperature distribution in the macrophage, the heat equation was solved over time. The general form of the heat equation is:

$$\rho C_p \frac{\partial T}{\partial t} = \nabla \cdot (k \nabla T) + Q$$

Additionally, the governing equations of momentum and mass conservation were included in the model to provide a comprehensive description of the system, even though fluid motion was not explicitly solved. These equations are given as:

a. Momentum conservation:

$$\rho\frac{\partial \boldsymbol{u}}{\partial t}+\rho(\boldsymbol{u}\cdot\nabla)\boldsymbol{u}=\nabla\cdot[-p\boldsymbol{I}+\boldsymbol{K}]+\boldsymbol{F}+\rho\boldsymbol{g}$$

where $\boldsymbol{u}$ is the velocity field (assumed stationary here), $\boldsymbol{p}$ is the pressure, $\boldsymbol{K}$ represents the viscous stress tensor, $\boldsymbol{F}$ includes external forces, and $\boldsymbol{g}$ is the gravitational acceleration.

b. Mass conservation:

$$\frac{\partial \rho}{\partial t}+\nabla(\rho\boldsymbol{u})=0$$

where the left-hand side describes changes in density over time, and the right-hand side accounts for mass flux divergence. Under the assumptions in this study, the medium is stationary, simplifying the equations.

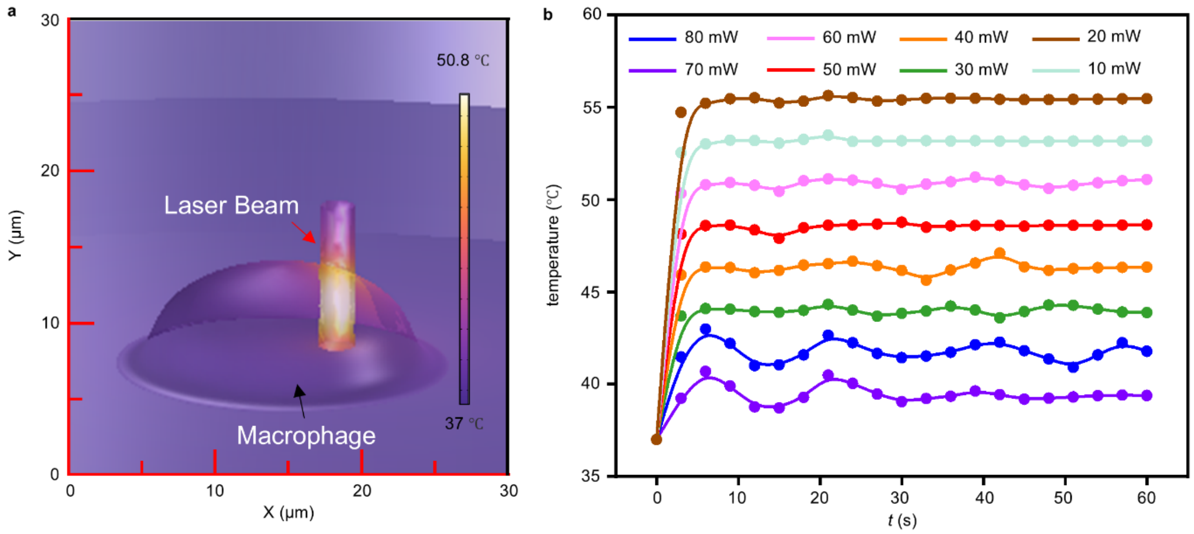

Figure S2. Simulation of photothermal effects of NIR light micro-irradiation on a single macrophage. (a) Model of the simulation used for the distribution and calculation of temperature field in a macrophage exposed to NIR light micro-irriadiation for 3 min. (b) Localized temperature changes in a macrophage after 3 min of irradiation with different laser powers.

1.3 Photodamage on a single macrophage

Photodamage to macrophages induced by NIR laser micro-irradiation was assessed by continuously irradiating the cells for 3 min, followed by cessation of the laser and subsequent observation for an additional 27 min, resulting in a total observation period of 30 min (Fig. S3). When the laser power was set to 60 mW, the stimulated cell showed intact cell morphology, with the continuous extension of the pseudopodia even after the cessation of the laser. On contrast, when the laser power was set to 80 mW, although the cell morphology remained intact during the 3-min micro-irradiation, it begun to shrinkage at t = 10 min and the cell morphology was completely broken at t = 30 min. This cellular morphology is indicative of a non-viable cell. The viability of macrophages following a 3-min laser micro-irradiation was assessed by determining the proportion of healthy cells relative to the total number of stimulated cells at each observation time point. The above experimental results demonstrate that the laser micro-irradiation at power levels below 60 mW does not induce photodamage in macrophages.

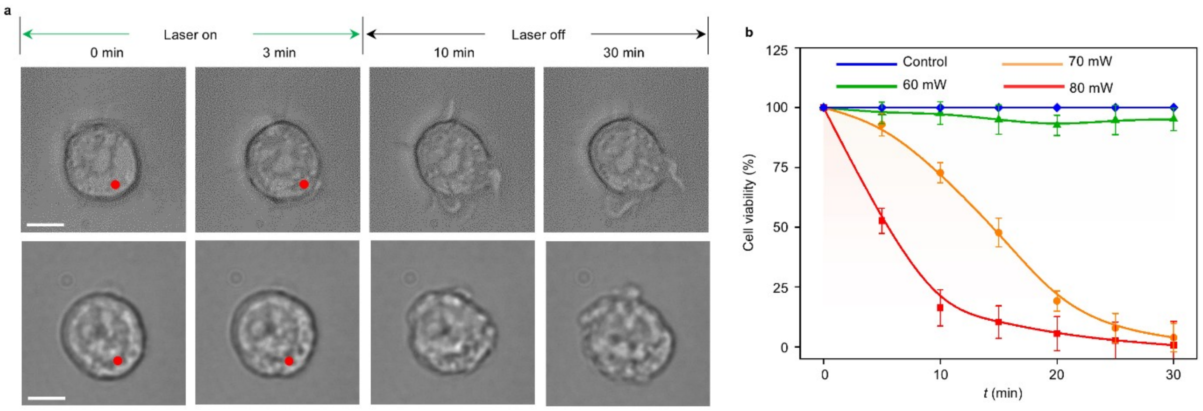

Figure S3. Photodamage on a macrophage at different laser power. (a) Time-lapse image sequences showing cell morphology after laser micro-irradiation for 3 min at laser power of 60 and 80 mW, respectively. Red dots indicate the position of light beam. Scale bar: 10 μm. (b) Cell viability of macrophages after laser micro-irradiation for 3 min at different laser powers. n = 15-20 cells.

To further examine the potential photodamage with prolonged light exposure, we observed the cell viability under 90-min light irradiation (Fig. S4) It should note the laser was initially focused on the cell edge to activate macrophages, and subsequently, it was continuously directed towards the extended pseudopodia instead of the cell body. This approach aligns with the actual experimental procedure. When the laser power is set as 60 mW, because the laser light is not directly focused on the cell body, the macrophage maintains contact morphology

during the 90-min light exposure, indicating the good cell viability. Since the initial 3-min irradiation at laser power of 80 mW has been shown to cause significant photodamage to cells (Fig. S3), extended light exposure leads to pronounced cell shrinkage and finally cell death. These findings suggest that when the laser power is below 60 mW, prolonged light exposure does not result in noticeable photodamage to the cells.

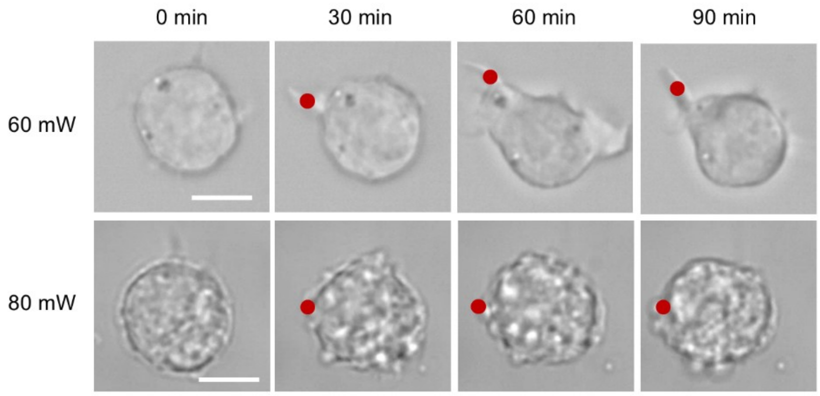

Figure S4. Time-lapse image sequences showing cell morphology under prolonged light exposure at laser power of 60 and 80 mW, respectively. Red dots indicate the position of light beam. Scale bar: 10 μm.

1.4 Analysis of the expression of thermosensitive cation membrane channel TRPM2 in macrophages

Transient receptor potential melastatin 2 (TRPM2) is a thermosensitive, Ca^{2+}-permeable cation membrane channel. The thermal activation of macrophages is reported to be tightly regulated by thermosensitive cation membrane channels, such as TRPM2.[2] These channels facilitate the influx of calcium ions and the depolarization of the plasma membrane, that are crucial for modulating macrophage activation.[3] The immunofluorescent staining analysis presented in Fig. S5 demenstrated that the macrophages used in this study largely expressed TRPM2, which may facilitate the optothermal activiation of macrophages via the NIR light micro-irridiation.

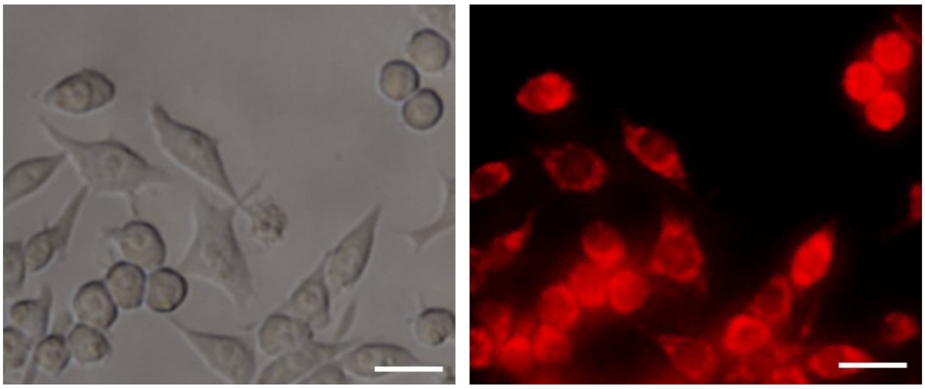

Figure S5. Immunofluorescent staining of TRPM2 in macrophages. Scale bar: 10 μm.

1.5 Estimation of the optical force exerted on the pseudopodia

When studying the optical forces applied on cellular pseudopodia, direct measurement of the force is technically impractical due to several inherent challenges. Pseudopodia are optically heterogeneous structures with irregular shapes and variable refractive indices, leading to unstable gradient forces and unreliable calibration. Additionally, their dynamic behavior, such as rapid extension or retraction, introduces noise and positional instability, making it difficult to quantify forces in real time. To estimate the optical force exerted on the pseudopodia, we used a 1-μm-diameter SiO_2 bead attached at the tip of pseudopodia as a force sensor (Fig. S6a). The SiO_2 bead, with uniform shape and refractive index, allow precise calibration of the optical trap's stiffness (k) through established methods (e.g., Brownian motion analysis). Once calibrated, the force exerted on the pseudopod can be indirectly calculated by tracking the microsphere's displacement (Δx) from the trap center using high-resolution detectors and applying the linear relationship $F=k\cdot\Delta x$. This approach can bypass the optical and dynamic complexities of pseudopodia. By slowly tethering the bead at a constant speed (10 μm/min) in the direction opposite to the cell body, the optical force exerted on the bead and the traction force generated by filopodia were analyzed. The measured optical forces at different laser power are shown in Fig. S6b, and are at the magnitude of pN level.[4,5]

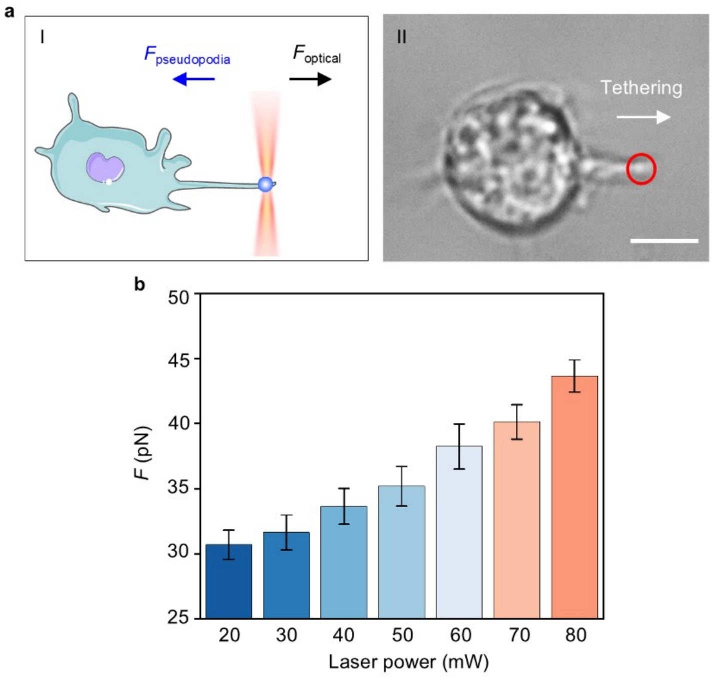

Figure S6. Estimation of the optical force exerted on the pseudopodia via attached bead. (a) Schematics of the setup (I) and experimental image (II) for tether extraction of pseudopodia via attached bead. Red circle in (II) indicates the 1-μm-diameter SiO_2 bead. Scale bar: 10 μm. (b) Measured optical force as function of laser power.

1.6 The phagocytosis of polystyrene (PS) nanoparticles and cellular debris of Hela cells by phagobot

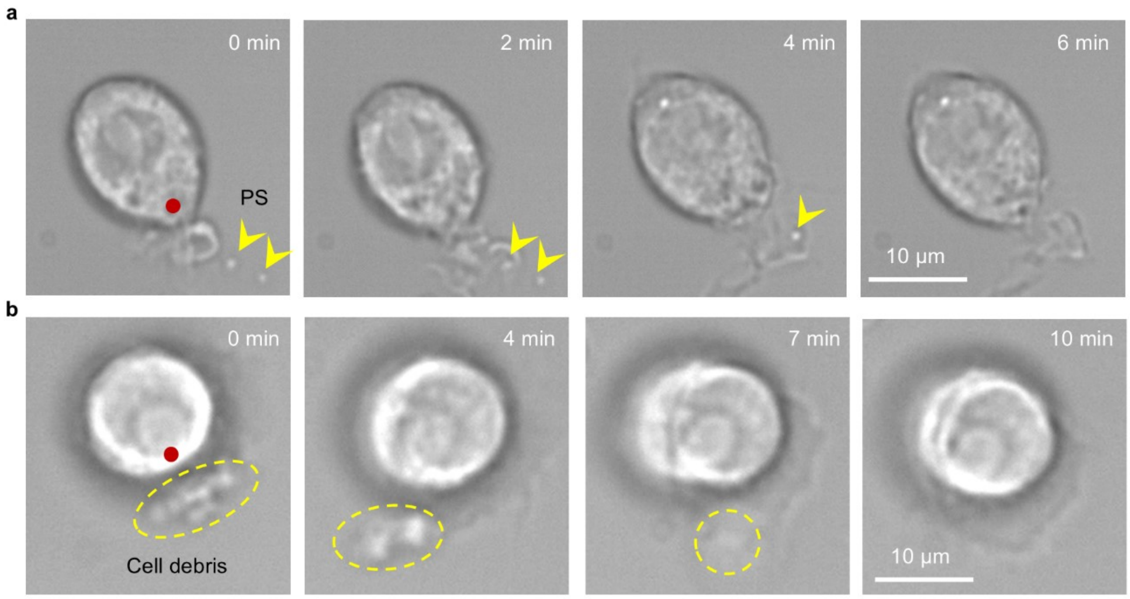

Figure S7. Time-lapse image sequences showing the phagocytosis process of (a) PS nanoparticles and (b) cellular debris of Hela cells by phagobot. Red dots indicate the position of light beam, yellow arrows indicate the PS nanoparticles and yellow dashed circles show the cell debris.

1.7 Analysis of simultaneous phagocytosis of multiple bio-threats by phagobot

Fig. S8 shows the simultaneous phagocytosis of multiple bio-threats by phagobot. Following the activation of the phagobot at $t = 0$ min (Fig. S8a), it was directed by optical forces to navigate specifically toward a cluster of *S. aureus* bacterial cells (indicated by white dotted box). At $t = 4$ min (Fig. S8b), the extended lamellar pseudopodia of the phagobot successfully reached the bacterial cluster and completed the phagocytosis of the bacteria by $t = 8$ min (Fig. S8c). Subsequently, the phagobot proceeded to navigate towards an additional five bacteria, which were dispersed in a parallel distribution and indicated by yellow arrows in Fig. S8d. Upon reaching these bacteria at $t = 16$ min (Fig. S8e), the phagobot initiated simultaneous phagocytosis of these multiple bio-threats, finishing the process by $t = 20$ min (Fig. S8f). These observations demenstrated the phagobot is capable of phagocytosing multiple bio-threats simultaneously.

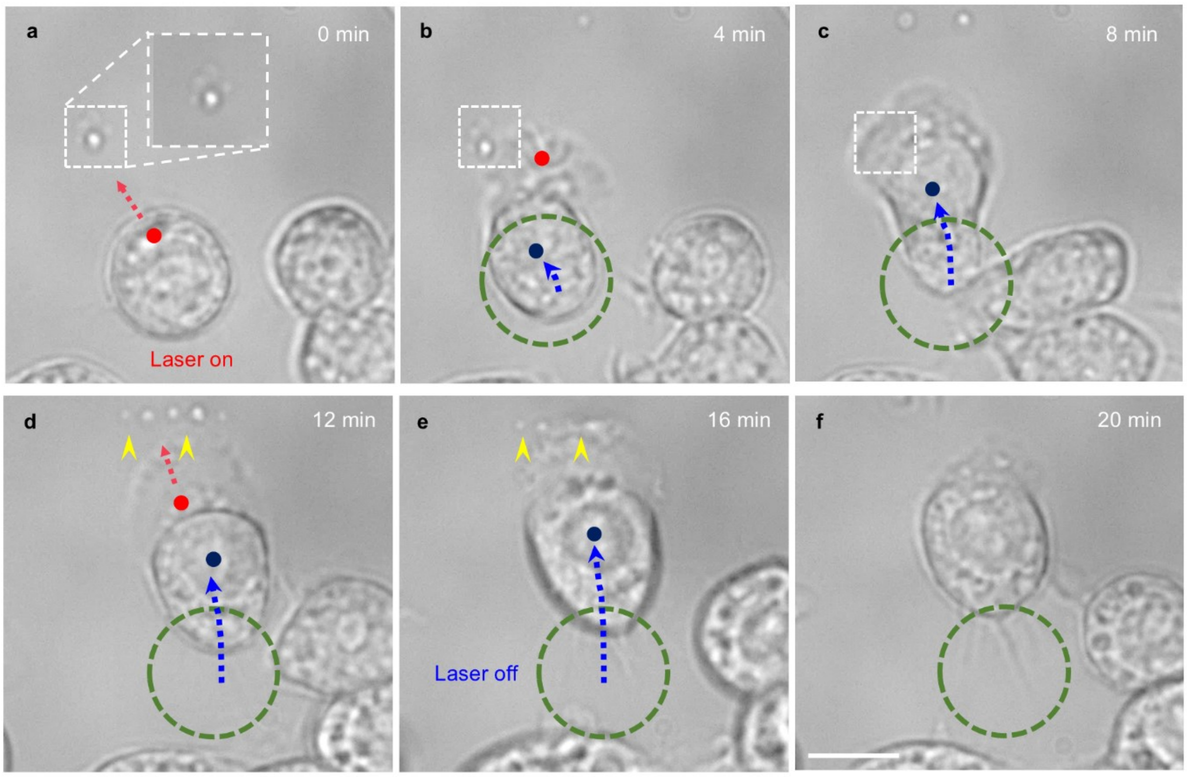

Figure S8. Simultaneous phagocytosis of multiple bio-threats by phagobot. White dashed boxes in (a-c) indicate the targeted multiple bio-threats of a cluster of *S. aureus* bacterial cells. Yellow arrows in (d-e) indicate the targeted multiple bio-threats of five parallelly distributed *S. aureus* bacterial cells. Red dots and red arrows indicate the position and moving direction of light beam, respectively. Green dashed circles and blue dashed lines indicate the initial position and the moving trajectory of phagobot, respectively. Scale bar: 10 μm.

1.8 Activiation of phagobot in vivo

Fig. S9 shows the *in vivo* morphological changes of macrophages under focused NIR light stimulation. Similar to macrophages cultured under in vitro 2D conditions, resting-state macrophages in in vivo 3D environments also exhibit a round morphology with a relatively smooth surface (t = 0 min). Upon stimulation within 3 min (t = 1 and 3 min) at the cell edge, the activated macrophages display a highly irregular morphology characterized by pseudopodia formation (indicated by yellow arrows). This irregular morphology becomes increasingly evident following the cessation of laser stimulation and continuous observation over the subsequent six min (t = 9 min), suggesting the successful activation of macrophages in vivo. Due to the differences in macrophage behavior between in vitro 2D culture conditions and in vivo 3D physiological environments, the in vivo morphological changes in activated macrophages are quite different. But the basic identification principles are the same, namely, the development of irregular morphology characterized by extended pseudopodia.

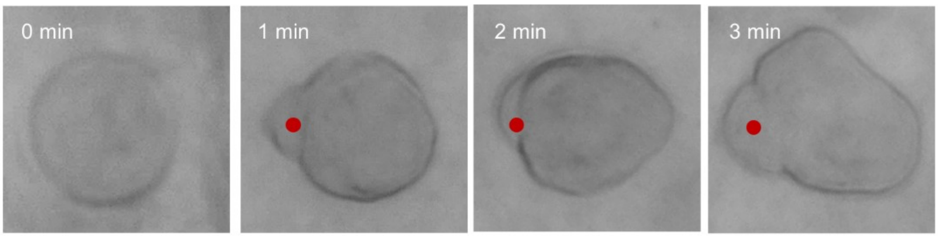

Figure S9. Time-lapse images showing the activation of a resting macrophage through focused NIR light (laser power: 80 mW) in vivo. Red dot indicates the position of NIR light beam, and yellow arrows indicate the extended filopodia. Scale bar: 5 µm.

1.9 In vivo bio-safety assay

Unlike in vitro experiments, in vivo biocompatibility is more complex and requires a broader evaluation. In addition to individual cell activity, it is crucial to consider potential tissue damage and the overall physiological state of the living organism. Therefore, we have monitored the heart rate of zebrafish during and after light exposure for in vivo biological safety assay of the phagobot system at organism levels (Fig. S10). The zebrafish heartbeat serves as a key indicator of systemic health, as cardiac abnormalities often reflect physiological stress or toxicity. The heart of transparent larval zebrafish can be clearly identified under optical microscopy, which is situated in proximity to the intestinal bulb (Fig. S10a). Red dot in Fig. S10a indicates the irradiation position of focused laser light. The heartbeat could be monitored through a charged-coupled device (CCD), which was connected to the computer for a real-time image capturing and video recording (Fig. S10b). The experiments are typically completed within 1 h; therefore, we monitored the heart rate during and following a 1-h irradiation of the intestinal bulb. Changes in heart rates when the zebrafish exposed NIR light at different laser power are shown in Fig. 5h.

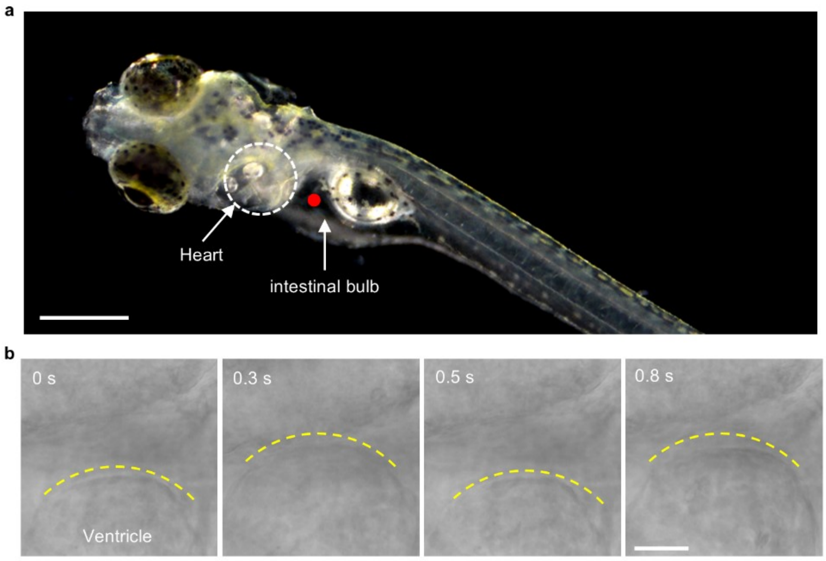

Figure S10. Characterization of changes in the heart rate of zebrafish. (a) Optical image indicating the location of heart in zebrafish. Scale bar: 500 μm. (b) Time-lapse image sequences showing the heart beating. Dashed yellow lines indicates the edge of heart ventricle. Scale bar: 50 μm.

Although the zebrafish remain alive at each laser power during prolonged light exposure for 1 h, bio-safety assessments at the cellular levels reveal that higher laser powers (above 90 mW) can induce significant photodamage to irriadated cells and tissues under the same

exposure duration (Fig. S11). Conversely, when the laser power is below 80 mW, no obvious photodamage to cells and tissues is observed, even with prolonged light exposure.

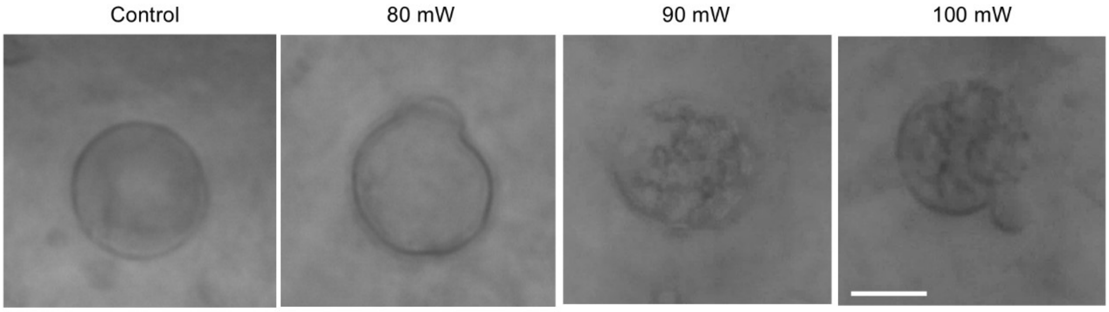

Figure S11. In vivo photodamage to cells at different laser power with prolonged light exposure for 1 h. Scale bars: 10 μm.

2. Description of Supplementary Videos

Video S1. Activation of a resting macrophage through NIR light micro-irradiation.

Video S2. Controllable steering of phagobot in vitro.

Video S3 Navigation of phagobot in vitro.

Video S4. Targeted phagocytosis by phagobot.

Video S5. Navigation of a phagobot in the intestinal lumen in vivo.

References

1. Krasnikov, I., Seteikin, A. & Bernhardt, I. Thermal processes in red blood cells exposed to infrared laser tweezers (λ = 1064 nm). *J. Biophotonics* **4**, 206-212 (2011).
2. Sumoza-Toledo, A., Penner, R. TRPM2: a multifunctional ion channel for calcium signalling. *J. Physiol.* **589**, 1515-1525 (2011).
3. Vig, M., Kinet, J.-P. Calcium signaling in immune cells. *Nat. Immunol.* **10**, 21-27 (2009).
4. Bornschlögl, T. et al. Filopodial retraction force is generated by cortical actin dynamics and controlled by reversible tethering at the tip. *Proc. Nat. Acad. Sci.* **110**, 18928-18933 (2013).
5. Leijnse, N. et al. Filopodia rotate and coil by actively generating twist in their actin shaft. *Nat. Commun.* **13**, 1636 (2022).
